# 1-[2,6-Dichloro-4-(trifluoro­meth­yl)phen­yl]-5-iodo-4-trifluoro­methyl­sulfinyl-1*H*-pyrazole-3-carbonitrile

**DOI:** 10.1107/S1600536809025082

**Published:** 2009-07-04

**Authors:** Ding-Xin Jiang, Han-Hong Xu

**Affiliations:** a Key Laboratory of Natural Pesticides and Chemical Biology of the Ministry of, Education, South China Agricultural University, Guangzhou 510642, People’s Republic of China

## Abstract

In the title compound, C_12_H_2_Cl_2_F_6_IN_3_OS, the dihedral angle between the planes of the benzene and pyrazole rings is 77.8 (2)°.  In the crystal, a short I⋯N contact of 2.897 (5) Å occurs.

## Related literature

For related structures containing phenylpyrazole, see: Shi *et al.* (2009 ▶); Tang, Zhong, Li & Hu (2005 ▶). Tang, Zhong, Lin, Hu & Shi (2005 ▶). 
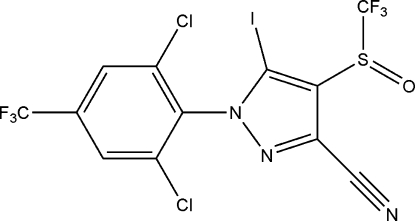


## Experimental

### 

#### Crystal data

C_12_H_2_Cl_2_F_6_IN_3_OS
*M* *_r_* = 548.03
Monoclinic, 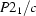

*a* = 9.5879 (3) Å
*b* = 13.7798 (4) Å
*c* = 14.3145 (5) Å
β = 107.400 (3)°
*V* = 1804.68 (10) Å^3^
*Z* = 4
Cu *K*α radiation
μ = 18.41 mm^−1^
*T* = 100 K
0.45 × 0.27 × 0.19 mm

#### Data collection

Oxford Diffraction Gemini (Cu) X-ray Ultra diffractometer
Absorption correction: analytical (*CrysAlis RED*; Oxford Diffraction 2006 ▶) *T*_min_ = 0.016, *T*_max_ = 0.213
8203 measured reflections
3132 independent reflections
3065 reflections with *I* > 2σ(*I*)
*R*_int_ = 0.041

#### Refinement

*R*[*F* ^2^ > 2σ(*F* ^2^)] = 0.041
*wR*(*F* ^2^) = 0.111
*S* = 1.13
3132 reflections
235 parameters
H-atom parameters constrained
Δρ_max_ = 0.77 e Å^−3^
Δρ_min_ = −1.25 e Å^−3^

### 

Data collection: *CrysAlis CCD* (Oxford Diffraction 2006 ▶); cell refinement: *CrysAlis RED* (Oxford Diffraction 2006 ▶); data reduction: *CrysAlis RED*; program(s) used to solve structure: *SHELXS97* (Sheldrick, 2008 ▶); program(s) used to refine structure: *SHELXL97* (Sheldrick, 2008 ▶); molecular graphics: *ORTEX* (McArdle, 1995 ▶); software used to prepare material for publication: *SHELXL97*.

## Supplementary Material

Crystal structure: contains datablocks I, global. DOI: 10.1107/S1600536809025082/bt2972sup1.cif

Structure factors: contains datablocks I. DOI: 10.1107/S1600536809025082/bt2972Isup2.hkl

Additional supplementary materials:  crystallographic information; 3D view; checkCIF report

**Fig. 1.**
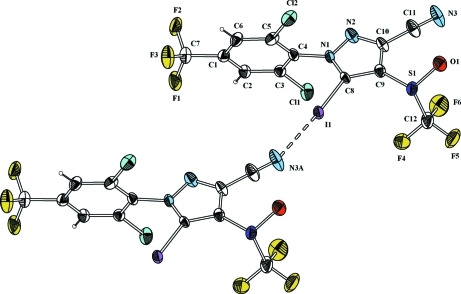
The molecular structure of the title compound with the atom-numbering scheme. Displacement ellipsoids are drawn at the 30% probability level and H atoms are shown as small spheres of arbitary radii.

Enhanced figure: interactive version of Fig. 1

## supplementary crystallographic information

### Comment

The molecular structure of title compound, C_12_H_2_Cl_2_F_6_IN_3_OS, is shown in Fig.1. The dihedral angle between the benzene ring and the pyrazole ring is 77.8 (2)°, while the corresponding ones in the two related compounds,*C*_12_H_4_C_l2_F_6_N_4_S (Tang,  Zhong,  Li   & Hu, 2005) and C_22_H_8_C_l4_F_6_N_8_S_2_ (Tang,  Zhong,  Lin, Hu & Shi,  2005), are 83.2 (1)° and 88.2 (1)°, respectively.

### Experimental

98% Fipronil (4.4 g, 10 mmol) was resolved in 40 ml chloroform in a 100 ml round bottom flask equipped with magnetic stirrer and a calcium chloride tube. Then iodine (3.6 g,14 mmol) was added into the solution. Half an hour later *tert*-butyl nitrite(1.43 g) was added into the solution and the mixture was heated under reflux for 2 h. Then it was left at room temperature overnight. The reaction mixture was filtered. Then the filtrate was evaporated *in vacuo*. The solid residue was purified by chromatography eluting using petroleum/ethyl acetate (4:1) and further recrystallized from toluene/hexane to afford colourless crystals. Yield: 4.70 g (86%).

### Refinement

H atoms   were included in calculated positions and treated in the subsequent refinement as riding atoms, with C—H = 0.93 Å and *U*_iso_(H) = 1.2*U*_eq_(C).

### Crystal data

**Table:**

C_12_H_2_Cl_2_F_6_IN_3_OS	*F*(000) = 1040
*M**_r_* = 548.03	*D*_x_ = 2.017 Mg m^−^^3^
Monoclinic, *P*2_1_/*c*	Cu *K*α radiation, λ = 1.54184 Å
Hall symbol: -P 2ybc	Cell parameters from 3132 reflections
*a* = 9.5879 (3) Å	θ = 4.6–67.0°
*b* = 13.7798 (4) Å	µ = 18.41 mm^−^^1^
*c* = 14.3145 (5) Å	*T* = 100 K
β = 107.400 (3)°	Prism, colorless
*V* = 1804.68 (10) Å^3^	0.45 × 0.27 × 0.19 mm
*Z* = 4	

### Data collection

**Table:**

Oxford Diffraction Gemini (Cu) X-ray Ultra diffractometer	3132 independent reflections
Radiation source: fine-focus sealed tube	3065 reflections with *I* > 2σ(*I*)
mirror	*R*_int_ = 0.041
ω and π scans	θ_max_ = 67.0°, θ_min_ = 4.6°
Absorption correction: analytical (*CrysAlis RED*; Oxford Diffraction 2006)	*h* = −10→11
*T*_min_ = 0.016, *T*_max_ = 0.213	*k* = −16→15
8203 measured reflections	*l* = −17→16

### Refinement

**Table:**

Refinement on *F*^2^	Primary atom site location: structure-invariant direct methods
Least-squares matrix: full	Secondary atom site location: difference Fourier map
*R*[*F*^2^ > 2σ(*F*^2^)] = 0.041	Hydrogen site location: inferred from neighbouring sites
*wR*(*F*^2^) = 0.111	H-atom parameters constrained
*S* = 1.13	*w* = 1/[σ^2^(*F*_o_^2^) + (0.0636*P*)^2^ + 4.9895*P*] where *P* = (*F*_o_^2^ + 2*F*_c_^2^)/3
3132 reflections	(Δ/σ)_max_ = 0.001
235 parameters	Δρ_max_ = 0.77 e Å^−^^3^
0 restraints	Δρ_min_ = −1.25 e Å^−^^3^

### Special details

**Table:**

Geometry. All e.s.d.'s (except the e.s.d. in the dihedral angle between two l.s. planes) are estimated using the full covariance matrix. The cell e.s.d.'s are taken into account individually in the estimation of e.s.d.'s in distances, angles and torsion angles; correlations between e.s.d.'s in cell parameters are only used when they are defined by crystal symmetry. An approximate (isotropic) treatment of cell e.s.d.'s is used for estimating e.s.d.'s involving l.s. planes.
Refinement. Refinement of *F*^2^ against ALL reflections. The weighted *R*-factor *wR* and goodness of fit *S* are based on *F*^2^, conventional *R*-factors *R* are based on *F*, with *F* set to zero for negative *F*^2^. The threshold expression of *F*^2^ > σ(*F*^2^) is used only for calculating *R*-factors(gt) *etc*. and is not relevant to the choice of reflections for refinement. *R*-factors based on *F*^2^ are statistically about twice as large as those based on *F*, and *R*- factors based on ALL data will be even larger.

### Fractional atomic coordinates and isotropic or equivalent isotropic displacement parameters (Å^2^)

**Table:**

	*x*	*y*	*z*	*U*_iso_*/*U*_eq_	
I1	0.32209 (3)	0.65606 (2)	0.31318 (2)	0.02325 (15)	
Cl1	0.49232 (14)	0.42087 (8)	0.14823 (9)	0.0336 (3)	
Cl2	0.37074 (16)	0.80256 (8)	0.09630 (9)	0.0364 (3)	
S1	0.70639 (13)	0.70720 (8)	0.47083 (8)	0.0257 (3)	
O1	0.8422 (4)	0.7630 (3)	0.4932 (3)	0.0360 (8)	
N1	0.5348 (4)	0.6307 (3)	0.1980 (3)	0.0215 (8)	
N2	0.6758 (4)	0.6311 (4)	0.1956 (3)	0.0312 (10)	
N3	1.0327 (6)	0.6582 (4)	0.3373 (4)	0.0474 (15)	
C1	0.1794 (5)	0.5669 (3)	−0.0465 (3)	0.0238 (10)	
C2	0.2659 (5)	0.4911 (3)	0.0021 (3)	0.0230 (9)	
H2A	0.2448	0.4273	−0.0183	0.028*	
C3	0.3855 (5)	0.5134 (3)	0.0823 (3)	0.0219 (9)	
C4	0.4171 (5)	0.6096 (3)	0.1111 (3)	0.0217 (9)	
C5	0.3304 (6)	0.6833 (4)	0.0603 (4)	0.0263 (10)	
C6	0.2084 (6)	0.6628 (3)	−0.0189 (4)	0.0279 (11)	
H6A	0.1480	0.7124	−0.0523	0.034*	
C7	0.0517 (6)	0.5443 (4)	−0.1351 (4)	0.0304 (11)	
C8	0.5210 (5)	0.6523 (3)	0.2870 (4)	0.0182 (9)	
C9	0.6611 (5)	0.6671 (3)	0.3484 (4)	0.0228 (10)	
C10	0.7517 (6)	0.6527 (3)	0.2873 (4)	0.0276 (11)	
C11	0.9089 (6)	0.6571 (4)	0.3153 (5)	0.0350 (14)	
C12	0.7590 (6)	0.5852 (4)	0.5261 (4)	0.0391 (13)	
F1	0.0028 (4)	0.4545 (3)	−0.1333 (3)	0.0488 (9)	
F2	0.0883 (4)	0.5527 (3)	−0.2176 (2)	0.0439 (8)	
F3	−0.0603 (4)	0.6042 (3)	−0.1430 (3)	0.0487 (9)	
F4	0.6443 (4)	0.5270 (2)	0.4989 (3)	0.0503 (9)	
F5	0.7988 (5)	0.5949 (3)	0.6227 (3)	0.0585 (11)	
F6	0.8683 (4)	0.5472 (3)	0.5007 (3)	0.0624 (11)	

### Atomic displacement parameters (Å^2^)

**Table:**

	*U*^11^	*U*^22^	*U*^33^	*U*^12^	*U*^13^	*U*^23^
I1	0.0165 (2)	0.0296 (2)	0.0220 (2)	−0.00092 (10)	0.00320 (13)	−0.00227 (10)
Cl1	0.0314 (6)	0.0221 (6)	0.0387 (7)	0.0056 (5)	−0.0026 (5)	0.0011 (5)
Cl2	0.0574 (8)	0.0171 (6)	0.0285 (6)	−0.0045 (5)	0.0033 (5)	−0.0014 (4)
S1	0.0264 (6)	0.0222 (6)	0.0230 (6)	−0.0021 (4)	−0.0008 (4)	−0.0002 (4)
O1	0.038 (2)	0.0290 (19)	0.036 (2)	−0.0106 (16)	0.0032 (16)	−0.0038 (16)
N1	0.0142 (18)	0.0247 (19)	0.0225 (19)	−0.0027 (16)	0.0008 (15)	−0.0014 (16)
N2	0.025 (2)	0.034 (2)	0.035 (2)	−0.0073 (17)	0.010 (2)	−0.0099 (19)
N3	0.021 (3)	0.064 (4)	0.058 (3)	−0.011 (2)	0.013 (2)	−0.035 (3)
C1	0.025 (2)	0.024 (2)	0.021 (2)	−0.0014 (19)	0.0050 (19)	−0.0004 (19)
C2	0.024 (2)	0.019 (2)	0.024 (2)	−0.0002 (18)	0.0043 (19)	−0.0038 (18)
C3	0.022 (2)	0.020 (2)	0.023 (2)	−0.0009 (18)	0.0050 (18)	−0.0004 (18)
C4	0.019 (2)	0.023 (2)	0.021 (2)	−0.0055 (18)	0.0039 (18)	−0.0053 (18)
C5	0.040 (3)	0.018 (2)	0.020 (2)	−0.002 (2)	0.007 (2)	−0.0022 (19)
C6	0.034 (3)	0.022 (2)	0.024 (2)	0.0029 (19)	0.002 (2)	0.0020 (18)
C7	0.025 (2)	0.031 (3)	0.030 (3)	0.003 (2)	0.000 (2)	−0.004 (2)
C8	0.010 (2)	0.018 (2)	0.025 (2)	0.0004 (15)	0.0038 (18)	0.0011 (16)
C9	0.021 (2)	0.017 (2)	0.027 (3)	−0.0013 (17)	0.002 (2)	−0.0014 (17)
C10	0.019 (3)	0.027 (3)	0.036 (3)	−0.0075 (18)	0.007 (2)	−0.009 (2)
C11	0.025 (3)	0.038 (3)	0.043 (3)	−0.008 (2)	0.012 (2)	−0.022 (2)
C12	0.034 (3)	0.029 (3)	0.042 (3)	−0.002 (2)	−0.008 (2)	0.009 (2)
F1	0.047 (2)	0.0386 (18)	0.0447 (19)	−0.0169 (16)	−0.0112 (15)	−0.0053 (15)
F2	0.0371 (18)	0.069 (2)	0.0202 (14)	0.0001 (16)	−0.0001 (12)	−0.0055 (15)
F3	0.0321 (17)	0.055 (2)	0.048 (2)	0.0130 (16)	−0.0052 (15)	−0.0163 (16)
F4	0.056 (2)	0.0313 (17)	0.053 (2)	−0.0147 (16)	0.0008 (17)	0.0124 (15)
F5	0.070 (3)	0.054 (2)	0.0337 (18)	−0.0074 (19)	−0.0127 (17)	0.0158 (16)
F6	0.047 (2)	0.049 (2)	0.084 (3)	0.0264 (18)	0.009 (2)	0.021 (2)

### Geometric parameters (Å, °)

**Table:**

I1—C8	2.051 (4)	C2—H2A	0.9300
Cl1—C3	1.728 (5)	C3—C4	1.394 (6)
Cl2—C5	1.731 (5)	C4—C5	1.375 (7)
S1—O1	1.463 (4)	C5—C6	1.392 (7)
S1—C9	1.764 (5)	C6—H6A	0.9300
S1—C12	1.863 (6)	C7—F1	1.327 (6)
N1—C8	1.353 (6)	C7—F3	1.332 (6)
N1—N2	1.363 (6)	C7—F2	1.333 (6)
N1—C4	1.438 (6)	C8—C9	1.382 (7)
N2—C10	1.330 (7)	C9—C10	1.419 (7)
N3—C11	1.134 (8)	C10—C11	1.440 (8)
C1—C2	1.384 (7)	C12—F6	1.316 (8)
C1—C6	1.384 (7)	C12—F4	1.322 (7)
C1—C7	1.508 (6)	C12—F5	1.327 (7)
C2—C3	1.393 (7)		
			
O1—S1—C9	108.7 (2)	C5—C6—H6A	120.8
O1—S1—C12	105.8 (2)	F1—C7—F3	107.4 (4)
C9—S1—C12	95.4 (2)	F1—C7—F2	106.6 (4)
C8—N1—N2	113.6 (4)	F3—C7—F2	106.9 (4)
C8—N1—C4	126.0 (4)	F1—C7—C1	112.2 (4)
N2—N1—C4	120.5 (4)	F3—C7—C1	112.0 (4)
C10—N2—N1	103.3 (4)	F2—C7—C1	111.4 (4)
C2—C1—C6	122.5 (5)	N1—C8—C9	106.3 (4)
C2—C1—C7	118.7 (4)	N1—C8—I1	122.4 (3)
C6—C1—C7	118.7 (5)	C9—C8—I1	131.3 (4)
C1—C2—C3	118.0 (4)	C8—C9—C10	104.2 (4)
C1—C2—H2A	121.0	C8—C9—S1	125.6 (4)
C3—C2—H2A	121.0	C10—C9—S1	129.9 (4)
C2—C3—C4	120.4 (4)	N2—C10—C9	112.6 (5)
C2—C3—Cl1	119.7 (4)	N2—C10—C11	120.1 (5)
C4—C3—Cl1	119.9 (4)	C9—C10—C11	127.2 (5)
C5—C4—C3	120.2 (4)	N3—C11—C10	178.4 (6)
C5—C4—N1	120.1 (4)	F6—C12—F4	109.8 (5)
C3—C4—N1	119.6 (4)	F6—C12—F5	108.9 (5)
C4—C5—C6	120.6 (4)	F4—C12—F5	108.8 (5)
C4—C5—Cl2	119.9 (4)	F6—C12—S1	112.3 (4)
C6—C5—Cl2	119.5 (4)	F4—C12—S1	109.1 (4)
C1—C6—C5	118.3 (5)	F5—C12—S1	107.9 (4)
C1—C6—H6A	120.8		
			
C8—N1—N2—C10	0.7 (6)	C2—C1—C7—F2	−94.0 (5)
C4—N1—N2—C10	−179.8 (4)	C6—C1—C7—F2	83.8 (6)
C6—C1—C2—C3	0.7 (7)	N2—N1—C8—C9	−0.7 (5)
C7—C1—C2—C3	178.4 (4)	C4—N1—C8—C9	179.9 (4)
C1—C2—C3—C4	−0.9 (7)	N2—N1—C8—I1	−179.5 (3)
C1—C2—C3—Cl1	177.4 (4)	C4—N1—C8—I1	1.1 (6)
C2—C3—C4—C5	−0.2 (7)	N1—C8—C9—C10	0.3 (5)
Cl1—C3—C4—C5	−178.5 (4)	I1—C8—C9—C10	178.9 (3)
C2—C3—C4—N1	175.7 (4)	N1—C8—C9—S1	174.4 (3)
Cl1—C3—C4—N1	−2.6 (6)	I1—C8—C9—S1	−6.9 (6)
C8—N1—C4—C5	75.7 (6)	O1—S1—C9—C8	−149.2 (4)
N2—N1—C4—C5	−103.6 (5)	C12—S1—C9—C8	102.1 (4)
C8—N1—C4—C3	−100.2 (6)	O1—S1—C9—C10	23.4 (5)
N2—N1—C4—C3	80.4 (6)	C12—S1—C9—C10	−85.3 (5)
C3—C4—C5—C6	1.6 (7)	N1—N2—C10—C9	−0.5 (6)
N1—C4—C5—C6	−174.3 (5)	N1—N2—C10—C11	178.0 (5)
C3—C4—C5—Cl2	−179.8 (4)	C8—C9—C10—N2	0.2 (6)
N1—C4—C5—Cl2	4.3 (6)	S1—C9—C10—N2	−173.6 (4)
C2—C1—C6—C5	0.7 (8)	C8—C9—C10—C11	−178.3 (5)
C7—C1—C6—C5	−177.1 (5)	S1—C9—C10—C11	7.9 (8)
C4—C5—C6—C1	−1.8 (8)	O1—S1—C12—F6	−50.8 (5)
Cl2—C5—C6—C1	179.6 (4)	C9—S1—C12—F6	60.4 (4)
C2—C1—C7—F1	25.4 (6)	O1—S1—C12—F4	−172.7 (4)
C6—C1—C7—F1	−156.8 (5)	C9—S1—C12—F4	−61.5 (5)
C2—C1—C7—F3	146.3 (5)	O1—S1—C12—F5	69.2 (5)
C6—C1—C7—F3	−35.9 (7)	C9—S1—C12—F5	−179.6 (4)
